# Structured Digital Self-Assessment of Patient Anamnesis Prior to Computed Tomography: Performance Evaluation and Added Value

**DOI:** 10.1007/s10916-020-01690-8

**Published:** 2021-01-28

**Authors:** M. Kopp, M. Wetzl, F. Geissler, J. P. Roth, R. Wallner, D. Hoefler, S. Faby, T. Allmendinger, P. Amarteifio, W. Wuest, A. Cavallaro, M. Uder, M. S. May

**Affiliations:** 1 Departement of Radiology, University Hospital Erlangen, 91054 Erlangen, Germany; 2 e.Bavarian Health GmbH, Erlangen, Germany; 3 Friedrich-Alexander University Erlangen-Nuremberg, Erlangen, Germany; 4 Siemens Healthcare GmbH, Forchheim, Germany; 5 Imaging Science Institute, Erlangen, Germany

**Keywords:** Digital medical history, Digital informed patient consent, Structured anamnesis, Mobile devices, Computed tomography

## Abstract

The aim of this study was to evaluate the performance of a tablet-based, digitized structured self-assessment (DSSA) of patient anamnesis (PA) prior to computed tomography (CT). Of the 317 patients consecutively referred for CT, the majority (*n* = 294) was able to complete the tablet-based questionnaire, which consisted of 67 items covering social anamnesis, lifestyle factors (e.g., tobacco abuse), medical history (e.g., kidney diseases), current symptoms, and the usability of the system. Patients were able to mark unclear questions for a subsequent discussion with the radiologist. Critical issues for the CT examination were structured and automatically highlighted as “red flags” (RFs) in order to improve patient interaction. RFs and marked questions were highly prevalent (69.5% and 26%). Missing creatinine values (33.3%), kidney diseases (14.4%), thyroid diseases (10.6%), metformin (5.5%), claustrophobia (4.1%), allergic reactions to contrast agents (2.4%), and pathological TSH values (2.0%) were highlighted most frequently as RFs. Patient feedback regarding the comprehensibility of the questionnaire and the tablet usability was mainly positive (90.9%; 86.2%). With advanced age, however, patients provided more negative feedback for both (*p* = 0.007; *p* = 0.039). The time effort was less than 20 min for 85.1% of patients, and faster patients were significantly younger (*p* = 0.046). Overall, the DSSA of PA prior to CT shows a high success rate and is well accepted by most patients. RFs and marked questions were common and helped to focus patients’ interactions and reporting towards decisive aspects.

## Introduction

Patient anamnesis (PA) is the information that a patient provides about their personal medical history. Many decisions and indications in clinical treatment pathways rely on PA, and most procedures performed upon the patient require informed consent. Nevertheless, informed patient consent and patient anamnesis obtained during patient-physician interactions are still mainly archived in hard-copy form and remain unavailable for further use. Digitization has the potential to increase the efficiency of healthcare systems [1]: improved software technology and mobile devices, meanwhile, can help to spread this technology into hospitals [2–5]. Particularly in radiology departments, high levels of digitization are already common and necessary; however, PA and patients’ current clinical problems are often incomplete, outdated, or inaccessible [6]. Self-assessment by the patient could be a strategy to overcome this lack of information quickly and effectively. The emerging availability of handheld devices in the medical environment enables patient interaction in the waiting room and allows the waiting time to become part of the examination.

Previous studies have yielded the numerous advantages of medical history-gathering systems [7–9], with key points of these systems including high patient and physician satisfaction and higher quantity and quality of requested information [10]. The benefits of these systems indicate that using them in hospitals could be appropriate for both the sometimes-impatient patients and the staff, who are often pressed for time. At the same time, the implementation of the systems could also aid staff in overcoming the lack of data availability and incompleteness of charts and could, in turn, improve the quality of procedures, diagnoses, and treatment [6]. Existing studies have examined the feasibility of tablet-based patient briefing in various contexts:, for example, prior to MRI examinations or in the pre-surgical assessment period [11–13]. Sufficient and complete gathering and presentation of PA is particularly important before computed tomography (CT), as radiation dose and iodinated contrast agent injections are ongoing concerns [14, 15]. According to the clinical task at hand, technological developments offer various options for radiation dose and contrast agent reduction. While such technologies are often complex in their interaction [16], and adverse effects from contrast media injections are relatively frequent and may be serious, they can nonetheless be safely avoided if the respective clinical information is completely available [17].

A digitized structured self-assessment (DSSA) of PA, utilizing specific questionnaires, can have numerous advantages compared to hard-copy-based notes. First, an adequate assessment of PA is often limited in clinical routines, as there is generally little time for patient-physician interactions. Second, verbally communicated PA is often only documented in fragments in an analog fashion; as such, it is not available for further purposes. Third, hard-copy-based data is rarely available for later usage on a different date or in an alternative place, and the notes may even be illegible. Finally, many patients recurrently pass CT examinations for follow-up reasons to monitor the dynamics of diseases under specific treatments: for example, in the case of malignant or infectious diseases. Prefilled digital inputs from previous examinations could, therefore, increase time-efficiency. In such cases, the patients would only refresh the information about their current clinical issues, while information about the chronic disease would be recorded only once. Above all, DSSA could facilitate access to complex PA and may focus the informed consent discussion on principal medical issues. Furthermore, structured access to relevant data has the potential to support and encourage the reporting process.

To our knowledge, there are, so far, no reports available in the literature about patient feedback and databank analysis after a DSSA of PA in the radiological environment. The purpose of this investigation was to evaluate the performance and explore the advantages of tablet-based DSSA of PA to facilitate informed consent discussion and the reporting process in clinical CT; as such, this study also aimed to fill the existing gap in the literature.

## Methodology

### Patient Client

**Fig. 1:**
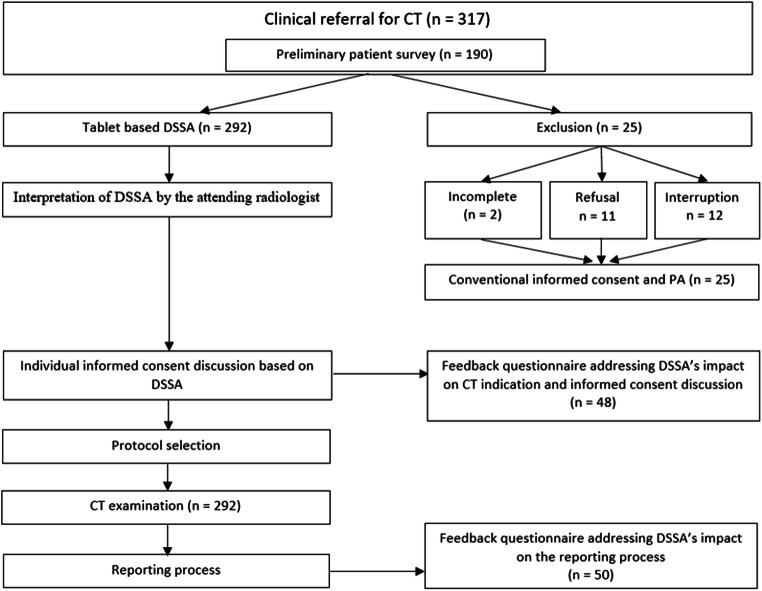
Study flow chart for tablet-based, digitized structured self-assessment (DSSA) of patient anamnesis (PA). The attending radiologist evaluated justified indication for computed tomography (CT) prior to study participation. On a mobile tablet device, the “red flags” were presented to the radiologist before informed consent was obtained. Before and during the interaction with the patient, all other digitized information from the self-assessment were also accessible in a structured fashion

This prospective, observational, single-center clinical study included consecutively referred patients with an elective indication for a CT examination. The various clinical problems included malignant tumor staging, follow-up examinations, infectious diseases, pain syndromes, auto-immune diseases, and chronic diseases. We used a dedicated software prototype of DSSA for PA (MEDePORT, e.Bavarian Health GmbH, Erlangen, Germany) on a tablet device (Surface Go, Microsoft Co., Redmont, WA, USA). Written informed consent was obtained from each patient prior to inclusion. Patients who were unable or refused to participate were excluded from the study and underwent conventional preparation for CT (Fig. 1). The extended anamnestic questionnaire was appended to the established brief questionnaire routinely obtained before patients’ written informed consent for CT.

Logging in to the dedicated patient client required the full name and birthdate of the patient. Alternatively, the radiographers or staff on the front desk could register the patients using the patient’s specific ten-digit identification number, either manually or via a barcode. After a short operational introduction, the patients processed the questionnaire, which comprised 67 questions, in the waiting room without further assistance. The majority of questions (*n* = 58) were collected in a structured way; free-text responses were limited to a minimum (*n* = 9). Unstructured free-text responses were only used for numeric data, such as laboratory values, and to allow for the input of responses beyond the structured chart, such as those involving rare symptoms, medication, and diseases. In the case of doubt or uncertainty, the patients could mark each question for further discussion during the subsequent individual patient briefing. Items from the DSSA were grouped into six main subjects: socio-cultural (e.g., lifestyle factors, family predisposition), medical (e.g., allergic reactions, current clinical symptoms), surgical (e.g., cholecystectomy, surgical bone repair), therapies (e.g., radio-chemotherapy, oncologic surgeries), imaging (e.g., prior imaging examination, complications due to imaging), and usability. We also asked the patients about their ratings for the questionnaire’s comprehensibility and the tablet’s usability on a 4-point Likert Scale (with answers ranging from “very good” – “good” – “average” – “poor”). Based on these answers, we created two subgroups: one for positive feedback (scores of “very good” and “good”) and one for negative responses (scores of “average” or “poor”). Moreover, the influence of patients’ ages on each subgroup was assessed. The patients estimated the duration of the DSSA on a 4-point Likert Scale (where answers ranged from “less than 10 minutes” – “10–20 min” – “20–40 min” – “more than 40 minutes”). Finally, the effect of patients’ age on their perception of the DSSA’s duration (< 20 min versus >20 min) was analyzed.

The patients returned the tablet devices to the radiographers after completion and moved on to the preparation room for a subsequent discussion with the radiologist. The waiting room was under passive surveillance by a study nurse for a subset of 190 patients to document rejections or interruptions during the DSSA. The on-premises software was installed on a local server, and all data were stored in a local relational database.

### Physician Client

**Fig. 2:**
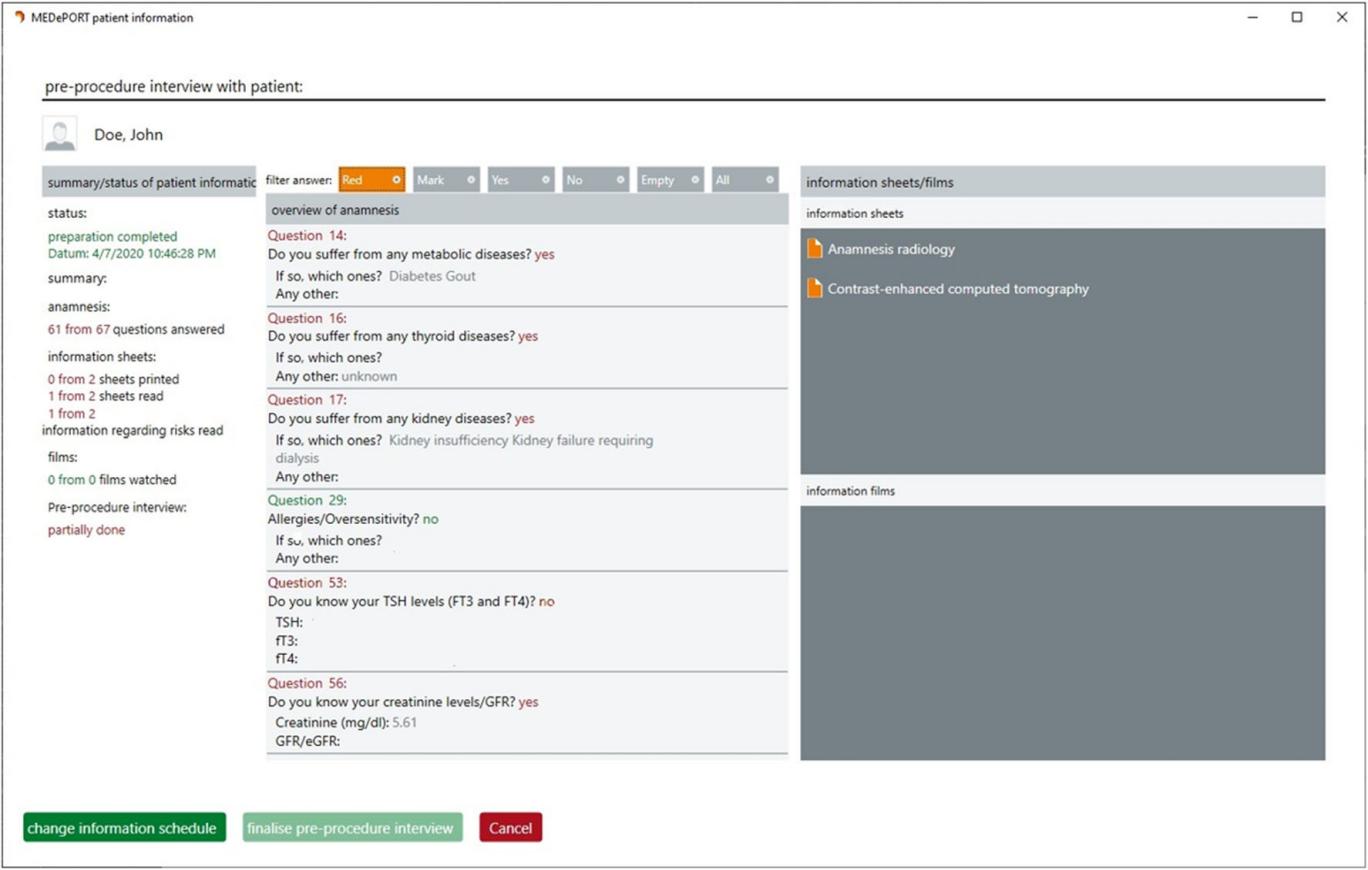
Structured overview of questionnaire content. This figure shows the structured RF answers on the overview of the physician client. Identified RFs for contrast enhanced computed tomography are highlighted in red-color font and include, for example, diabetes, high creatinine levels, unknown thyroid disease, and unknown thyroid values. After reviewing the RF content, the physician could review marked questions together with the patient. The client could also display unanswered (“Empty”) or “All” questions

The attending radiologist was able to log in to the physician client with a username and password on an advanced mobile tablet device (Surface Pro, Microsoft Co., Redmont, WA, USA) or any desktop computers at our institution. Updates of the patient worklist were automatically received from the radiological information system (RIS). The software allowed for the manual assignment of the dedicated anamnesis questionnaire for this study to the patient account. One or more out of 803 informed consent templates from all medical fields were available in different languages. “Native CT” or “Contrast-enhanced CT” templates were assigned to all patients in the study. Potentially critical answers addressing CT indication, contrast media injection, and current symptoms were identified by consensus of a working group of senior radiologists (over ten years of experience) prior to the study (Table 1). This information, along with the “red flag” (RF) characteristics, was presented as a structured overview on the first page of the physician client whenever the patient completed the questionnaire (Fig. 2). Other filters that could be applied to the results of the questionnaire: were 1) “Marked” questions; 2) all “Yes” questions; 3) all “No” questions; 4) “Unanswered” questions; 5) an overview about “All” questions. The attending physician confirmed the justifying indication for a CT examination based on the information provided by the RIS on the results of the DSSA and the informed consent discussion. In the case of agreement, a digital document in PDF format had to be digitally signed on the tablet by both the patient and the physician to complete the legal aspects of the informed consent. The CT examination was then performed with regard to all available clinical data following our institutional standardized operating procedures. Standard picture archiving and communicating system (PACS) workstations with full access to the physician client was used for reporting.

**Table 1:** “Red flag” questions. Answers that activate a “red flag” were printed in bold and automatically structured on the physician client’s front page

“Red flag” topics	“Red flag” answers
Do you suffer from pain?	Yes (severe pain intensity 8–10 on a visual analog scale)
	No
Do you have claustrophobia?	Yes No
Have you ever had side effects after the administration of a contrast agent?	Yes
	- mild: nausea, itching, erythema
	- moderate: vomiting, shortage of breath, wheals
	- severe: allergic shock, coma, cardiac arrest
	–
	- other symptoms (free-text field)
	No
Are there any known underlying thyroid diseases?	Yes
	- hyper-function
	- hypo-function
	- thyroid nodes
	- tumor
	- thyroiditis
	- goiter
	- other pathologies (free-text field)
	No
Do you have scheduled radio-iodine therapy?	Yes
	No
Are current thyroid hormone levels available?	Yes
	- Thyroid-stimulating hormone [mU/l]
	- free Triiodothyronine [ng/dl]
	- free Thyroxine [ng/dl]
	No
Do you take thyroid medication?	Yes
	- Thyroxin
	- Thiamazol
	- Carbimazole
	- Thiouracil
	- Sodium perchlorate
	- other medications (free-text field)
	No
Are there any known underlying kidney diseases?	Yes
	- impaired renal function
	- inflammation
	- tumor
	- urinary stones
	- blood in urine
	- status after kidney surgery
	- other diseases (free-text field)
	No
Are current creatinine values available?	Yes
	- creatinine value [mg/dl]
	No
Are there any known underlying metabolic diseases?	Yes
	- Diabetes mellitus
	- Gout
	- other diseases (free-text field)
	No
Do you take diabetes medication?	Yes
	- Insulin
	- Metformin containing drugs
	- other medications (free-text field)
	No
Do you suffer from an infectious disease?	Yes
	- Hepatitis
	- HIV/AIDS
	- Tuberculosis
	- other diseases (free-text field)
	No
Are you pregnant or breastfeeding?	Yes
	No

### Clinical Evaluation

Existing clinical information provided by the referring physicians was classified as “background information,” “clinical problem,” and “diagnosis” for each case. In addition, structured feedback from the radiologists about the direct impact of PA on the clinical process of informed consent and reporting was collected by implementing a dedicated form. This form was completed for a randomly selected subgroup of patients after the informed consent discussion (*n* = 48) and after reporting (*n* = 50). Each questionnaire covered the issues “content” and “impact on the clinical process,” both of which were rated on a 3-point Likert scale (with answers ranging from “crucial” – “partly relevant” – “not relevant”). The specific type of relevant “content” was additionally assessed in the context of the following subgroups: social, family, symptoms, lifestyle, chronic disease, prior surgery, medication, prior examination, and laboratory values.

### Computed Tomography

The examinations were performed using a 64-slice computed tomography scanner with a rotation time of 0.33 s (SOMATOM go.Top, Siemens Healthcare GmbH, Forchheim, Germany). All contrast medium enhanced examinations were performed using bodyweight adapted with intravenous injection of 350 mg Iodine/ml (Iomeprol, Iomeron 300, Bracco Imaging, Milan, Italy) via power injector (Accutron CT-D, MEDTRON AG, Saarbrücken, Germany).

### Data Processing and Statistics

The recorded patient data was exported as an extensible markup language file (.XML) and processed with the Excel software package (Microsoft Co., Redmond, WA, USA). Further statistical analysis was performed using the software package SPSS Statistics Version 21 (International Business Machines Corporation [IBM], Somers, NY, USA). Normal distribution was assessed using the Kolmogorov-Smirnov and Shapiro-Wilk tests. Normally distributed data were presented as mean ± standard deviation. The median and interquartile ranges were provided if no normal distribution was assumed. The normally distributed data were compared using paired t-tests if normal distribution was not assumed, the Mann-Whitney U test was used for further analysis. The significance level was defined as *p* < 0.05.

## Results

### Patient Client

The total patient collective consisted of 317 patients (44.2% female, 55.8% male). The mean age was 55.3 ± 15.0 years, while BMI was 25.7 ± 6.6. The subgroup covered by nurse surveillance (*n* = 190) exhibited a low prevalence of refusal (5.8%) or interruption (6.4%), and a total of 294 patients consented to study participation. Two patients answered less than ten questionnaire items. We excluded these patients from the study due to limited digital data availability. All patients with significant problems concerning the DSSA performed a conventional informed consent procedure.

Concerning patients’ educational qualifications, 17.4% of patients declared a university degree; 16.4% had a university-entrance diploma; 60.6% held a certificate of secondary education, while 2.4% had no degree. Most patients passed a prior CT examination (89.8%) or reported a previous contrast agent injection (39.1%).

**Fig. 3:**
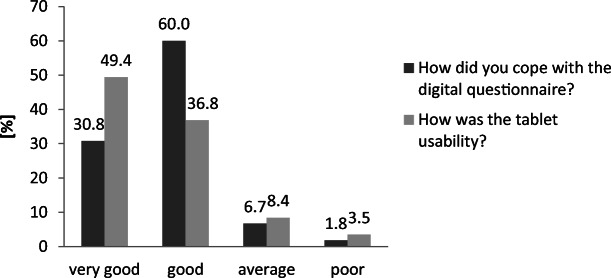
Patient feedback for comprehension and usability of digitized structured self-assessment (*n* = 292). A large number of patients expressed high satisfaction values for questionnaire comprehensibility and tablet usability

**Fig. 4:**
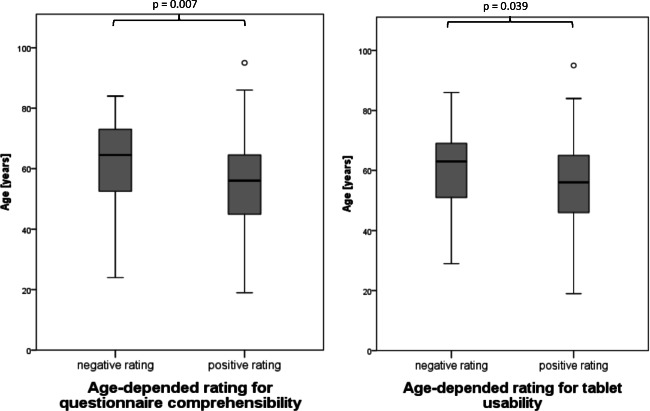
Age-dependent patient feedback for questionnaire comprehensibility and tablet usability. Patients with advanced age rated more negatively. Boxplot-and-whisker diagram: boxes show medians, first-quartile, and third-quartile boarders. Whiskers display minimum and maximum values within 1.5 times the interquartile range. Outliers are marked as °

**Fig. 5:**
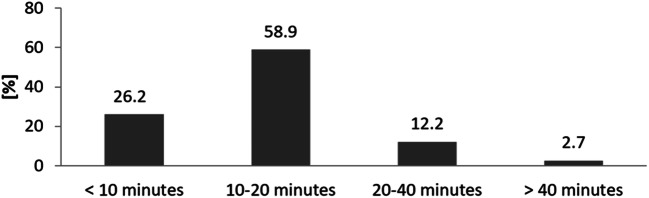
Estimated duration of digitized structured self-assessment (*n* = 292). The majority of patients finished the questionnaire in less than 20 min (84.8%)

**Fig. 6:**
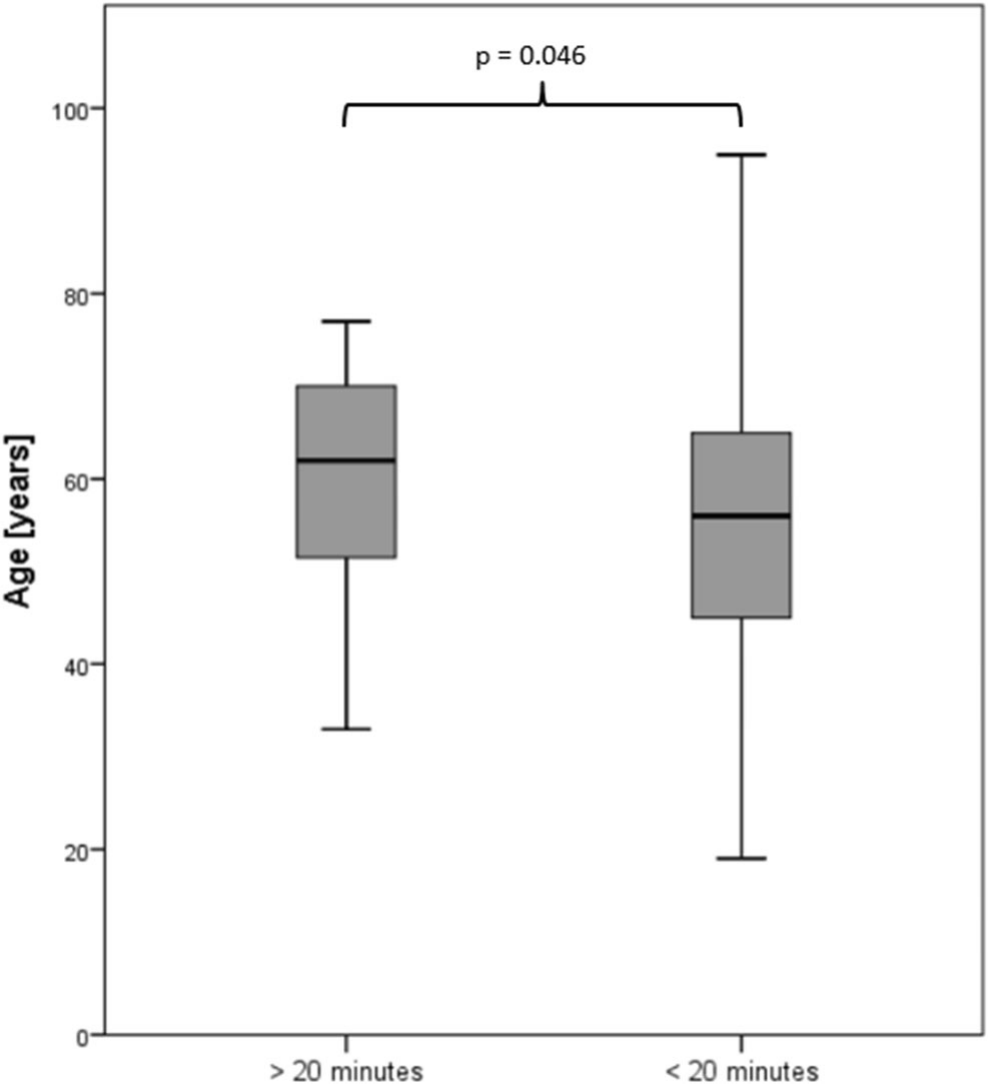
Comparison of age-dependent duration of digitized structured self-assessment. Patients who needed more than 20 min for completion were significantly older than those who needed less than 20 min. Boxplot-and-whisker diagrams: boxes show medians, first-quartile, and third-quartile boarders. Whiskers display minimum and maximum values within 1.5 times the interquartile range

Almost every patient was able to finish the usability evaluation (96.9%). Questionnaire comprehensibility (90.8%) and tablet usability (86.2%) were rated positively by the majority of patients (Fig. 3). Negative ratings about comprehensibility were obtained from significantly older patients (median age 64.5; 52.3–73.0 years) compared to positive ratings (median age 56; 45.0–65.0 years; *p* = 0.007). Patients who rated tablet usability as negative (median age 60.6; 51.0–69.1 years) were older than patients with a positive rating (median age 54.4; 45.3–65.0 years; *p* = 0.039; Fig. 4). The greater part of the patients (85.1%) completed the questionnaire in less than 20 min; a minority of 14.9% needed more than 20 min for completion. Patients who required less than 20 min (median age 56; 45.0–65.0 years) were significantly younger than patients with longer completion times (median age 62.0; 51.1–71.2 years; *p* = 0.046; Figs. 5 and 6).

### Physician Client

There was a high prevalence of “marked questions” (26%), especially for the topics of underlying malignant diseases (15.8%), scan range (14.5%), and already-performed CT examinations (13.2%). RFs were prevalent in 69.5% (Table 2) of patients. Absolute contraindications were rare, with 1.0% of patients declaring a thyroid hyper-function and 0.3% scheduled for radio-iodine therapy. None of the patients were breastfeeding or pregnant. Relative contraindications were more common: missing creatinine values (33.3%), kidney diseases (14.4%), thyroid diseases (10.6%), metformin (5.5%), claustrophobia (4.1%), allergic reactions to contrast-agents (2.4%), and pathological TSH values (2.0%).

**Table 2:** Prevalence of highlighted anamnestic information. “Marked” questions were highlighted by the patients during self-assessment in case of doubts or uncertainties. “Red flags” were automatically highlighted by the system. These items were addressed explicitly during the individual patient briefing prior to the examination

	total (%)
Study collective	n = 292
Total “marked” questions	n = 76 (26%)
Most frequently “marked” questions	
- Do you have a history of malignant diseases?	n = 12 (4.1%)
- Which body region will be examined?	n = 11 (3.8%)
- Do you have a history of CT examinations?	n = 10 (3.4%)
Total “Red flag” questions	n = 203 (69.5%)
Severe pain symptoms	n = 10 (3.4%)
Claustrophobia	n = 12 (4.1%)
History of contrast agent side effects	n = 7 (2.4%)
Thyroid pathology	n = 31 (10.6%)
Scheduled or current radio-iodine therapy	n = 1 (0.3%)
Intake of thyroid medication	n = 25 (8.6%)
- Levothyroxine	n = 20 (6.8%)
- Iodide	n = 4 (1.4%)
- Carbimazol	n = 1 (0.3%)
Kidney disease	n = 42 (14.4%)
Pathologic creatinine values >1.2 mg/dl	n = 10 (3.4%)
Missing creatinine values	n = 97 (33.3%)
Metabolic disease	n = 40 (13.6%)
- Diabetes mellitus	n = 25 (8.6%)
- Metformin	n = 16 (5.5%)
Infectious diseases	n = 14 (4.8%)
- HIV/AIDS	n = 3 (1.0%)
- Tuberculosis	n = 3 (1.0%)
- Hepatitis (A-D)	n = 4 (1.4%)
Pregnancy and/or breastfeeding	n = 0 (0%)

### Clinical Evaluation

“Background information” and information about “diagnosis” were often incomplete in the RIS (73.5%). We found incomplete details on the “clinical problem” prior to CT in 29.8% of cases. Meanwhile, all three subtypes of clinical information were missing in only 1.0% of all cases. In such cases, the CT indication had to be justified after additional consultation of the referring physician. Clinical indication for CT mainly included staging or follow-up examination of malignant disease (69.2%), inflammatory disease (15.5%), acute pain syndromes (10.3%), imaging of benign tumors (2.4%), anatomic pre-surgical evaluation (1%), screening for lung fibrosis (0.7%), and detection of kidney stones (0.7%).

Structured feedback from the attending radiologists about the feasibility and chances of DSSA was available for 48 cases after the informed consent discussion and 50 cases after reporting.

The radiologists rated the “content” of the DSSA as “crucial” for most of the cases after the informed consent discussion (85.4%) and in many cases after the reporting process (58%). In only a minority of cases, the DSSA provided no relevant “content” for informed consent discussion (4.2%) and reporting process (16%). The most relevant topic for the informed consent discussion and the reporting process was “chronic disease” (39.1% and 64.6%). Furthermore, “prior examinations” were helpful in around a third of all cases during both the informed consent discussion and the reporting process. The relevance of the current “symptoms” was rated substantially higher for the reporting process (56.3%) than for informed consent discussion (30.4%). In comparison, “medication” appeared more relevant for the informed consent discussion (32.6%) compared to the reporting process (20.8%). A crucial “impact on the clinical process” was rated in more cases during the reporting process (42.0%) compared to the informed consent discussion (31.3%; Table 3). DSSA was deemed irrelevant for 56.3% during the informed consent discussion and 40% during the reporting process.

**Table 3:** Illustration of radiologist feedback about digital structured self-assessment (DSSA) of patient anamnesis (PA). Feedback was available for the issues “content” and “impact on the clinical process.” The radiologists evaluated the extent to which the contents of DSSA contributed relevant information to informed consent discussion and the reporting process and how they rated the impact on the clinical process

	Informed consent discussion (*n* = 48)		Reporting process (*n* = 50)	
Content				
- Crucial	85.4%		58%	
- Partly relevant	10.4%		26%	
- Not relevant	4.2%		16%	
Type of enhanced content	Chronic disease	39.1%	Chronic disease	64.6%
	Prior examinations	37%	Symptoms	56.3%
	Medication	32.6%	Prior examinations	35.4%
	Symptoms	30.4%	Prior surgery	33.3%
	Laboratory values	17.4%	Medication	20.8%
	Prior surgery	17.4%	Lifestyle	20.8%
	Lifestyle	10.9%	Laboratory values	0%
	Social	4.3%	Social	0%
	Family	2.2%	Family	0%
Impact on clinical process				
- Crucial	31.3%		42.0%	
- Partly relevant	12.4%		18.0%	
- Not relevant	56.3%		40.0%	

### Computed Tomography

The most common contrast-enhanced examinations were chest CT (33.7%), chest-abdomen CT (32.5%), neck-chest-abdomen CT (17.2%), abdomen CT (9.8%), and whole-body CT (2.0%). The most common unenhanced examinations were chest CT (16.2%), paranasal sinus CT (8.4%), and unenhanced abdomen CT (1.3%). Overall, the injection of iodine-containing contrast agent was necessary for 79.3% of patients.

## Discussion

Our study has shown that a tablet-based DSSA of PA is feasible, highly accepted by the majority of patients, and indicates a reasonable time effort of below 20 min for most patients. There was significantly more negative patient feedback from older patients. “Red flags” were highly prevalent and could be retrieved automatically from digital PA. Moreover, structured PAs helped complete the medical background information prior to CT, which was incomplete in three-fourths of examinations, and aided the radiologist in interpreting the images during reporting.

The comprehensive questionnaire about PA is capable of recording and structuring specific medical history and assessing social background anamnesis (e.g., family status, educational achievements), which may be helpful for proper preparation of informed consent discussion and use of language. Most of our patients had experience in the CT environment, which could have contributed to sufficient questionnaire comprehension. This is underlined by the very positive patient feedback for DSSA, which is in line with some of the study results from Schlechtweg et al., who analyzed patient briefing with mobile tablet computers before magnetic resonance imaging (MRI) [11]. The time effort to complete our extended questionnaire with 67 items is also minimally comparable to that of other studies in the radiology environment. In our opinion, the required time effort of about 20 min for most patients is reasonable and could be significantly reduced by tailoring the questionnaires to the current clinical situation, especially in follow-up examinations and specific CT indications. Despite the overall positive patient feedback, however, our results also indicate that older patients have more difficulties in using and understanding DSSA, which is illustrated by increasingly negative patient feedback and more extensive time effort for older patients. This may be due to reduced fine motor skills, minor adaptation to a digitized environment, and the increasing health impairments in older patients. These findings are unlike those of a study by Alikhani et al.: they evaluated mobile patient briefing with 12 questions prior to CT and found no age-dependent differences concerning the duration of patient briefing [18]. We assume that this higher negative patient feedback among older patients in our study may be related to the more than fivefold number of questions that we assessed, and, hence, a fatigue effect in this sub-collective. Future digital questionnaires could overcome this problem with a better preselection of questions, ideally related to the clinical task. Moreover, further technical improvement may better facilitate the use of mobile tablet computers. The highest convenience is expected if DSSAs are completed on private devices, likely already at home, where relatives may help to cope with digital workflow challenges. The mainly positive feedback from the attending radiologists about informed consent workflow and the reporting process is in line with the findings of Haussen et al., who evaluated a smartphone platform for electronic informed consent in acute stroke care and found a streamlined consenting process [19]. Nevertheless, none of these studies reported details about individual PA, patient feedback, or impact on patient care.

The in-depth analysis of our database indicates that about one-fourth of the patients needed clarification about several questions. Additionally, we found a high prevalence of RF due to various issues. Thus, DSSA should not be interpreted as a substitute, but rather a catalyst for patient-physician interactions. Clinically relevant information was incomplete for many patients from the traditional RIS interface, even while most cases were non-emergencies. This can potentially cause inappropriate examination settings or the misinterpretation of imaging findings, leading to severe patient harm or disadvantages. Time-consuming inquiries are needed to avoid these critical situations, which we found are strongly reduced using PA data from DSSA. Besides making individual PA immediately available, the presented software also allows for a retrospective analysis of the entire collective in the structured database. The creation of such a database is hoped to impact future research and, as a result, wider clinical routines.

## Limitations

When analyzing this study, some limitations have to be considered. First, all answers in the questionnaire were filled out voluntarily, and, therefore, not every question was answered by every patient. More specific and shorter questionnaires would be less time consuming and could contribute to increased patient satisfaction and questionnaire completion rates. Second, the patient feedback is likely influenced by not only age but also educational status and professional experience, all of which vary from region to region. Thus, our results may be only partly transferable to other radiology institutions. Furthermore, our results concerning DSSA duration are based on individual estimates by the patients. A timestamp function was not available for the current software solution. Third, some patients are unable to read or have impaired fine-motor skills. It also appears inappropriate to perform DSSAs in emergency settings. Excluding these patients leads to somewhat unbalanced patient collectives. Fortunately, the error rate was relatively low in our collective, especially considering the advanced median age. Fourth, it remains unclear if the patients’ responses in a semi-private environment were truthful and medically correct. The discrepancy between almost 80% contrast-enhanced CT examinations in our department and the anamnestic 40% of prior contrast injections raises doubts, at the very least, about the state of medical knowledge in our patient collective. However, this problem inheres to every anamnestic assessment. Lastly, it must be noted that spreading digital applications into hospitals will increase dependency on server technology and power supply and will, in turn, raise the need for data security solutions.

## Conclusion

Our study indicates that DSSAs of PA are largely accepted by patients and can be performed within reasonable timeframes prior to CT. Prevalence of RFs and marked questions are high, and the assessed data has an important impact on informed consent discussion and reporting. Therefore, the automatic and structured presentation of prioritized data helps to focus on the most critical health issues. Moreover, thorough database analysis may help to increase knowledge about the health issues of the local patient cohort, something which should be focused upon in future projects.
